# Vanilla and Vanillin as Probable Causes of Hypersensitivity Reactions and Vaping Cytotoxicity

**DOI:** 10.3390/cimb48080771

**Published:** 2026-07-29

**Authors:** Kinga Lis

**Affiliations:** Department of Allergology, Clinical Immunology and Internal Medicine, Ludwik Rydygier Collegium Medicum in Bydgoszcz, Nicolaus Copernicus University in Torun, ul. Ujejskiego 75, 85-168 Bydgoszcz, Poland; kinga.lis@cm.umk.pl

**Keywords:** vanilla, vanillin, hypersensitivity, allergy, cytotoxicity, e-cigarettes

## Abstract

Vanilla and vanillin are the most popular flavorings added to processed foods, medications, cosmetics, and e-cigarette liquids. Vanilla owes its unique aroma to vanillin. Both vanilla and vanillin can cause hypersensitivity reactions, probably type IV. Clinically, hypersensitivity to vanilla and/or vanillin most often manifests as a limited rash (of various types) accompanied by itching and/or swelling. Long-term exposure to vanillin or its intermediates may be a factor in occupational sensitization. Although hypersensitivity to vanilla/vanillin appears to be rare, it is worth considering in the diagnostic process, especially in non-obvious situations when identifying the triggering allergen is difficult. Regardless of the likely allergic hypersensitivity to vanilla and vanillin, there are also hypotheses suggesting that vanillin and other flavorings and aromas found in e-cigarette liquids may have cytotoxic effects in people who use electronic cigarettes (vaping). Toxic reactions caused by inhaling vapors of these additives may affect the bronchial tree and other distant organs. This hypothesis, although new and relatively poorly researched, may have significant implications for public health, including for future generations, especially considering that vaping is a popular form of nicotine delivery, particularly among adolescents and young adults.

## 1. Introduction

Vanilla is one of the most commonly used flavoring agents for food, cosmetics and medicines [1]. Vanilla came to Europe from South America, from which it was brought by Spanish conquistators at the turn of the 16th and 17th centuries [2]. The Maya and Aztecs were the first to develop the curing of vanilla pods. This happened long before Europe became fascinated by this delicate aroma. Early South American cultures used cured vanilla beans to perfume their temples, while green beans were used in medicine, to treat people bitten by poisonous insects and to heal wounds [3]. In the 18th century, European pharmacists also appreciated the medicinal properties of vanilla [4]. In 1721, it was included for the first time in the Pharmacopoeia of the Royal College of Physicians of London (Latin: Pharmacopoeia Collegii Regalis Medicorum Londinensis) [5]. Despite the numerous health-promoting properties of vanilla [2] being known for centuries, nowadays it is mainly used as a flavoring additive to food, medicines (mainly for children), cosmetics and industrial products (e.g., household chemicals, e-cigarettes).

## 2. Vanilla and Vanillin—Similarities and Differences

**Figure 1:**
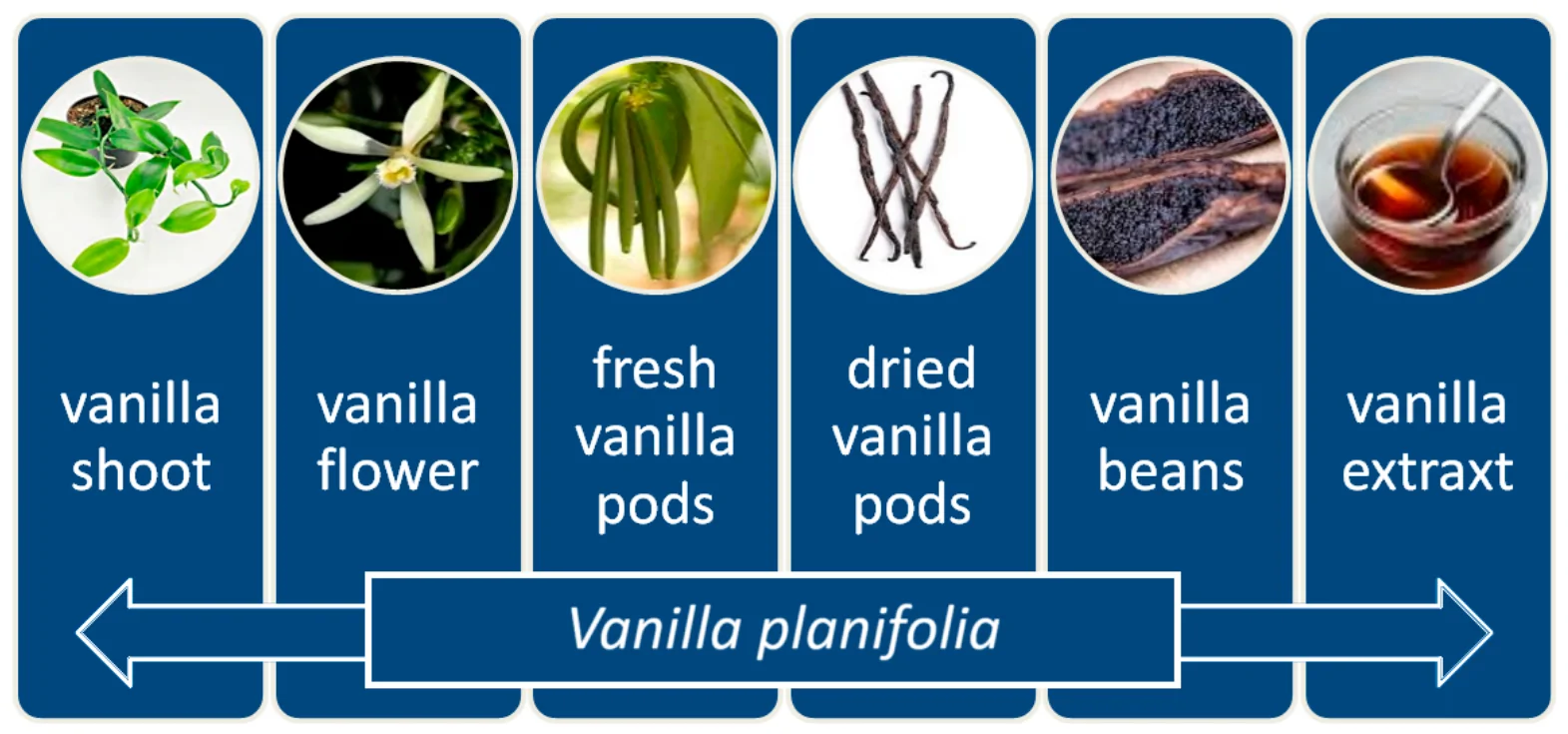
*Vanilla planifolia*.

Vanilla is extracted from the unripe pods of the Vanilla vine (Latin: *Vanilla* spp.), a plant of the orchid family (Latin: *Orchidaceae*), mainly *Vanilla planifolia* (Latin: *Vanilia planifolia*, *V. planifolia*) (Figure 1) [1,6].

Vanilla grows naturally in the tropical forests of South and Central America. The ancient Aztecs, who inhabited what is now Mexico, were one of the first civilizations to use vanilla, obtained from the pods of the wild *Vanilla planifolia*, to tone down the bitterness of raw chocolate [7]. Currently, natural vanilla, from *Vanilla planifolia,* is available as dried pods, powder, oil (liquid extract), or vanilla sugar [8].

**Figure 2:**
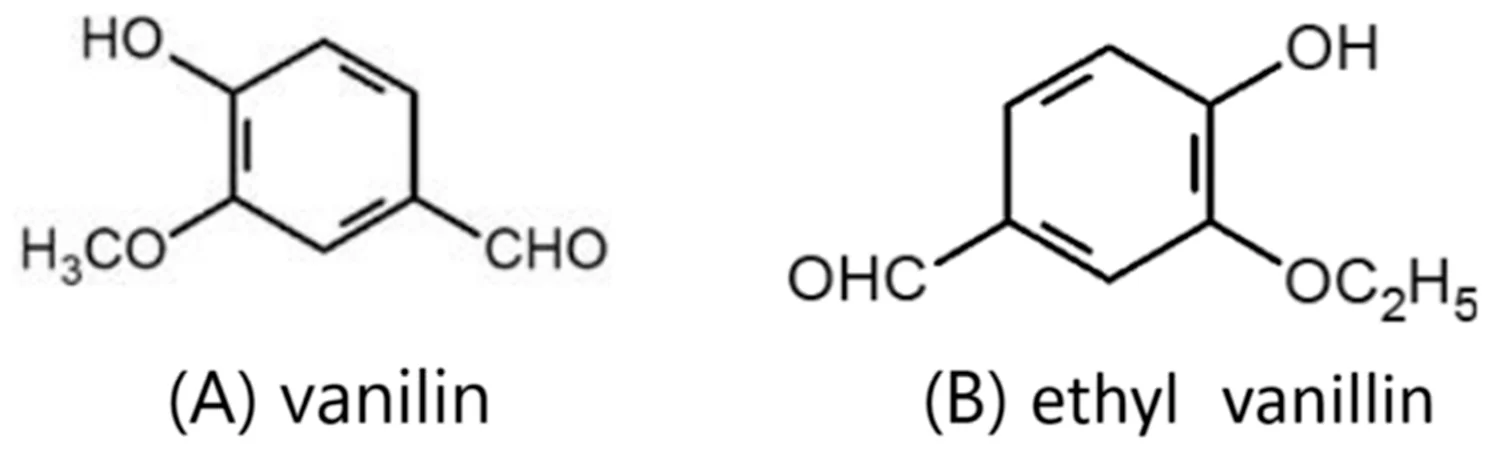
Structural formulas of vanillin (**A**) and ethyl vanillin (**B**) [9,10].

Dried vanilla beans contain over 300 chemical compounds, over 170 of which are volatile aroma components. The main vanilla aroma components from vanilla beans are vanillin, vanillic acid, vanillyl alcohol, p-hydroxybenzaldehyde, p-hydroxybenzoic acid, and p-hydroxybenzyl alcohol. Vanillin (3-methoxy-4-hydroxybenzaldehyde) constitutes approximately 2% of the total plant material in *Vanilla planifolia* [9,10]. Aromatic compounds, primarily vanillin (Figure 2A) and its derivatives, mainly ethyl vanillin (Figure 2B), give vanilla extracts a specific, unique aroma.

Vanillin for industrial use comes from two sources: produced by extraction from natural raw materials (natural vanillin) and produced by chemical synthesis (synthetic vanillin) [1,9,10]. Currently, only three (*Vanilla planifolia*, *Vanilla tahitensis*, and *Vanilla pompona*), out of approximately 100 *Vanilla* spp. varieties, are commercially important. The most valuable is vanilla extracted from the pods of *V. plantifolia* [2]. Obtaining natural vanilla from this plant is very difficult because *V. plantifolia* is pollinated exclusively by Melipona bees, which are found only in tropical and subtropical countries of Africa, Asia, Australia, and America [11,12]. Therefore, until the first half of the 19th century, Mexico was the only vanilla producer in the world. It was not until the development of hand-pollination techniques [2,6,13,14] that the plant was cultivated in various regions of the world [15]. Natural vanilla is one of the three most expensive spices in the world (the other two being saffron and cardamom) [1,2,6,16].

*Vanilla planifolia* is not the only natural source of vanillin. This aromatic substance occurs in many different sources of plant origin (e.g., lignins, which are waste products during wood processing and cellulose production) [1,9,10,17].

Synthetic vanillin can be produced by chemical synthesis from phenol or guaiacol [1,17] or by bioconversion of ferulic acid, obtained from rice bran, or bioconversion of eugenol, by strains of *Corynebacterium* or *Pseudomonas* bacteria [18]. There are also known less conventional sources of this aroma in the production of synthetic vanillin, such as cow dung [19,20]. Vanillin is classified as natural only if both the substrate and the method used to isolate vanillin are natural. Vanillin is defined as synthetic if its extraction is performed using inorganic chemicals or is synthesized through chemical processes [21]. Synthetic vanillin is much more commonly used than natural vanillin. Consumers typically do not distinguish between natural vanilla and vanillin [17,21]. Both vanillin and vanillin are used as flavoring additives in food products (approx. 60% of vanillin consumption), medicines (approx. 7%), cosmetics (approx. 33%), and various industrial products [17,21]. It is worth noting that in the pharmaceutical industry, vanillin is used both as a flavoring and as a substrate for drug synthesis [17].

## 3. Hypersensitivity to Vanilla and/or Vanillin

**Figure 3:**
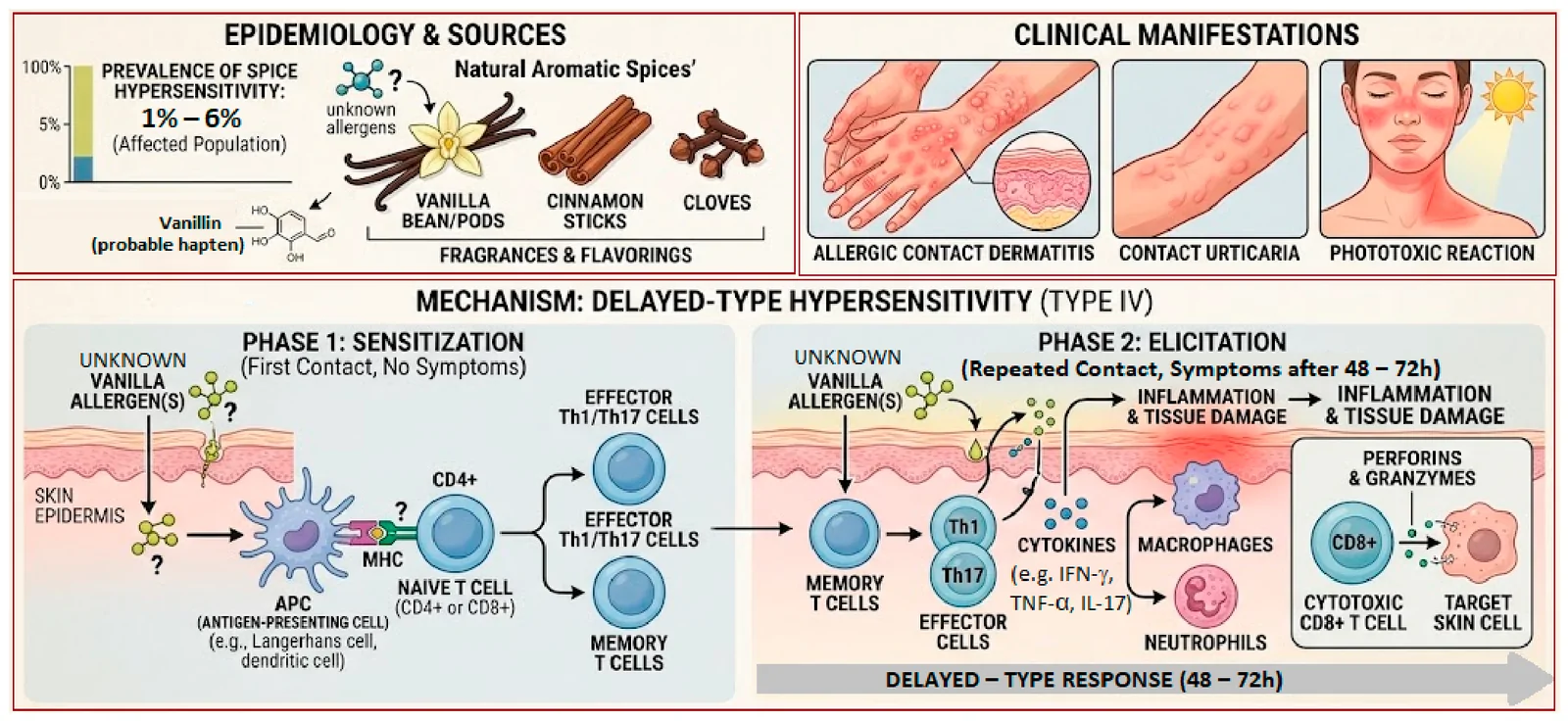
Vanilla hypersensitivity: epidemiology, clinical manifestations, probable mechanism.

Hypersensitivity to natural aromatic spices such as vanilla, cinnamon, and cloves is estimated to affect between 1% and 16% of the unselected population [22]. The most common clinical symptoms include allergic contact dermatitis, contact urticaria, and photoallergic reactions. The mechanism by which vanilla induces hypersensitivity reactions has not yet been elucidated. The spectrum of clinical symptoms suggests delayed-type hypersensitivity (type IV) or pseudoallergic reactions. Vanilla allergens are unknown, but it is suspected that vanillin (the main flavoring component of vanilla) may act as a low-molecular-weight contact allergen (hapten), similar to other low-molecular-weight chemicals such as preservatives, dyes, or flavors [23,24,25] (Figure 3).

### 3.1. Hypersensitivity to Vanilla and/or Vanillin—Clinical Cases

#### 3.1.1. Contact Allergen

The first case of a contact dermatitis hypersensitivity reaction to vanilla was reported by Hutchinson in 1893 [26]. Many authors refer to this description as the first published case of a contact hypersensitivity reaction caused by skin contact with food containing an allergen, in this case vanilla [27,28,29]. It is also believed that Hutchinson, by describing this clinical case, was the first to draw attention to the fact that food additives, including spices, can cause hypersensitivity reactions, and the occurrence of clinical symptoms does not have to be related to the consumption of the allergen [27,28,29].

In 1995, Ferguson and Beck [30] described recurrent inflammation of the lips and perioral dermatitis in a 13-year-old girl, caused by a protective lip balm flavored with vanilla. The child underwent patch tests with 10% vanilla and with the lipstick she was using, and both gave positive results. This seems to confirm that the cause of the dermatitis was the vanilla contained in the cosmetic. The only uncertainty is that it was not specified whether the lipstick contained natural vanilla or vanillin. This is important because natural vanilla extract from the plant *Vanilla* spp. Apart from vanillin, it contains numerous other substances (such as tannins, polyphenols, free amino acids, resins, acids, ethers, alcohols, acetals, heterocyclic compounds, phenols, hydrocarbons, esters and carbonyls) [15], which may also cause hypersensitivity.

Another case of skin hypersensitivity to vanilla contained in cosmetics was reported in 1941 [31]. Leggett [31] described a patient in whom the use of a vanilla-flavored shampoo caused persistent itching of the scalp and face. Leggett’s description [31] is referred to in a case of scalp hypersensitivity, which was reported in detail on the blog “www.homiyo.com” VANILLA AS A SKIN IRRITANT Posted by Dr James|27 May 2020” (accessed on 18 August 2023) [32]. Here we describe the clinical history of a patient who experienced persistent itching of the scalp after using a homemade balm containing vanilla essence (other ingredients of the balm are: quinine, lavender spirit and rectified spirit). The balm had been used by the patient many times before and did not cause any adverse symptoms. Approximately twenty-four hours after applying the cosmetic, the patient experienced intense itching of the scalp, which gradually spread to the forehead, behind the ears and down to the neck area. The patient, wanting to alleviate the symptoms, rubbed the scalp with vanilla, because he was convinced of its soothing and cooling effect and, as a supporter of homeopathy and natural medicine, had used vanilla oils for this purpose before. However, this action did not bring the expected result by the patient, on the contrary, it exacerbated the itching and swelling of the face and eye area occurred. Since the patient had used vanilla many times before, he was very surprised by this situation and decided to carry out a self-diagnosis. For this purpose, he rubbed the skin of his forearm with the vanilla essence he had been using. After 24 h, a rash appeared on the skin of his forearm, accompanied by annoying itching. The rash disappeared spontaneously and recurred on average every 6 h for about 14 consecutive days. Similarly to what had happened earlier, on the scalp. The patient mentioned that the itching of the scalp lasted for a very long time and was bothersome especially at night, because it disturbed his sleep. The patient also presented numerous scratches on the skin of his scalp, face and forearms, which coincided with areas that had been in contact with vanilla essence or a hair conditioner containing it. It seems that from the given description it can be concluded that the patient did indeed experience symptoms of a hypersensitivity reaction after skin contact with vanilla essence. Interestingly, this patient had also consumed vanilla many times in his life, because he really liked the taste and aroma, but it had never led to any symptoms of hypersensitivity before [32].

#### 3.1.2. Airborne Allergen

The clinical hypersensitivity to vanillin reported by Kojder et al. [33] highlights another interesting aspect of vanillin allergy. These authors described a case of contact dermatitis after airborne exposure to vanillin. The described patient developed hypersensitivity symptoms in the form of redness, urticaria, and swelling of the skin of the face and neck 48 h after exposure to sprayed vanilla extract. According to van Assendelft [34], exposure to vanillin by inhalation may cause bronchospasm, which was confirmed in controlled, double-blind challenge tests with vanillin.

#### 3.1.3. Occupational Allergen

Available data indicate that vanilla, vanillin, and its derivatives can also cause occupational allergies, particularly occupational contact dermatitis resulting from irritation [35]. Case reports of contact dermatitis have been published in individuals professionally involved in vanilla cultivation or processing of vanilla or vanillin. The occupational groups most at risk of contact hypersensitivity to vanilla and/or vanillin include confectioners, bakers, producers of sweets, beverages, or cosmetics to which vanilla or vanillin is added during the production process, as well as individuals professionally involved in the cultivation of *Vanilla* spp. or the synthesis of vanillin [36,37,38]. Wang et al. [37] described an interesting case of atopic dermatitis that developed in over 50% of the workforce (53 of 105 workers; men and women) at a vanillin production plant. Almost all of the workers who developed skin lesions had been employed at the plant for more than a year. The researchers conducted numerous patch and intradermal tests with chemicals used throughout the production line, as well as with the initial substrate (petroleum derivatives) and the final product (vanillin). Interestingly, positive reactions were observed with each chemical used in the vanillin synthesis process, except for the initial substrate and the final product (vanillin). The authors of this study concluded that vanillin applied to the skin itself is not irritating, but it cannot be ruled out that vanillin, present in large quantities in factory dust, combined with other components of this dust, including coarse dust, may contribute to the induction or exacerbation of inflammatory skin lesions [37].

### 3.2. Hypersensitivity to Vanilla and/or Vanillin—Studies on Allergenicity

There are only few studies on the allergenicity of vanilla and/or vanillin. Moreover, for *Vanilla* spp., for example, they do not only concern the flavoring substance itself but concern different parts of these plants. This situation significantly complicates drawing consistent conclusions on the sensitization potential and possible mechanisms of hypersensitivity to vanilla and/or vanillin.

In 1961, Hjorth [39] assessed the potential of *Vanilla planifolia* and *Vanilla tahitensis* pods to cause skin irritation. The subjects underwent a series of patch tests using vanilla pod pulp (5 mm long per test). The test results were read three times, i.e., after 48, 96 and 120 h after applying the vanilla pulp to the skin. None of the patients had a positive reaction at any of these three test points. One patient had a very late hypersensitivity reaction (on day 9 after applying the test paste to the skin), which appeared at the site where the vanilla pulp was applied to the skin. In the further part of the study, the same nine patients with eczema were subjected to patch tests with an alcoholic vanilla extract (10% *w*/*w*) and an acetone vanilla extract (10% *w*/*w*). The plant sources of both extracts were *Vanilla planifolia* and *Vanilla tahitensis*. A positive reaction to the alcoholic vanilla extract was observed in 7 and to the acetone extract in 1 of 9 patients tested [39]. Hjorth [39] continued the study in a similar scheme and examined 73 patients diagnosed with hypersensitivity to Peruvian balsam. Thirty-four of them had positive reactions to both vanilla species. In conclusion, Hjorth [39] did not completely rule out the role of vanilla in causing or exacerbating dermatitis, but he does not believe that this is a very common phenomenon and rather concerns people who are allergic to vanillin regardless of its source. For example, an allergy to Peruvian balsam may be related to the vanillin it contains [39].

### 3.3. Hypersensitivity to Vanilla and/or Vanillin—Connection with Allergy to Peruvian Balsam

The association of hypersensitivity to Peruvian balsam with hypersensitivity to vanillin seems to be justified. Peruvian balsam is a pathological, resinous secretion obtained by mechanical damage to the bark of the balsam tree (*Myroxylon balsamum Harms var. pereirae Leguminosae*) from the legume family, growing in South America [40]. Although vanillin constitutes only 1.3% of the content of Peruvian balsam, it is estimated that hypersensitivity to vanillin is responsible for the delayed reaction in about 2.6% of patients allergic to this resin, both by contact and ingestion [39,41,42,43]. It is also important that Peruvian balsam is a mixture of various substances and other active compounds contained in it can also cause hypersensitivity reactions of varying degrees of intensity. This significantly complicates the unambiguous identification of the causative factor, which may or may not be vanillin. For example, in a study conducted in Poland [44], in a group of 22 patients with propolis-induced dermatitis, 19 had positive patch test results not only with propolis but also with Peruvian balsam. Two patients who were simultaneously allergic to propolis and Peruvian balsam were also hypersensitive to vanillin. In this group of patients, hypersensitivity to clove oil was also common [44]. The main component of clove oil is eugenol [45], which is one of the substrates for the production of vanillin by oxidation. It should be noted, however, that the results of patch tests performed on a group of 102 patients with hypersensitivity to balsam of Peru by Hausen et al. [46] with various aromas and aroma mixtures that are commonly used in patch tests confirm that allergy to balsam of Peru does not necessarily mean allergy to vanillin and may be caused by any other component of this natural resin. These observations draw attention to the possible cross-reactivity between different spices and flavorings, which is still poorly studied and is a challenge for both diagnostics and therapy in people allergic to spice ingredients and food additives, including flavoring substances.

Kanny et al. [47] analyzed the association of various flavoring substances with the occurrence of atopic dermatitis in children. Eleven children under five years of age with severe atopic dermatitis were administered a dietary questionnaire (with the help of their caregivers) and double-blind oral challenges with natural vanilla (50 mg), synthetic vanillin (12.5 mg), and balsam of Peru (225 mg), a natural resin whose main flavor is vanillin. The substances to be tested were selected based on the results of the dietary questionnaire, which indicated a high daily intake of natural vanilla and/or vanillin, which was predominant among other natural and artificial flavorings consumed by children. Nine out of 11 children developed eczematous reactions following the challenge, and one of them also developed Quincke’s edema. In two children, all the test results were negative. In all children with positive results, it was recommended to eliminate food containing vanilla or vanillin. This procedure resulted in a reduction in skin lesions in six of them. The authors [47] also pointed out that the risk associated with the consumption of flavoring agents, including vanillin, is often not sufficiently appreciated and the traditional approach to considering these substances as safe or neutral may be harmful to consumers.

### 3.4. Hypersensitivity to Vanilla and/or Vanillin—Difficulties in the Analysis of Published Data

The analysis and evaluation of reported cases of vanilla and/or vanillin hypersensitivity are somewhat difficult. This is due to, among other things, the small number of available data, the frequent confusion between vanilla and vanillin allergy, the lack of a clear distinction between the origin of the allergen (natural or synthetic). Some of the available reviews are also based on retrospective studies, including the analysis of patch test results, different batches, and different fragrance mixtures. Most of the available data are more than 10 years old and there are no new case reports and studies of hypersensitivity to vanilla, vanillin and other flavoring agents commonly used in food, medicines, cosmetics and other everyday products.

In this context, an interesting example may be the description of a case of anaphylaxis reported by Jappe et al. [48], which occurred in a man after accidental ingestion of peanuts, and was published under the extremely misleading title “Anaphylaxis to vanilla ice cream: a near fatal cross-reactivity phenomenon” [48]. In the report of this case, the authors only mentioned that the patient, currently diagnosed with shock after accidental ingestion of peanuts, had anaphylaxis after ingestion of vanilla ice cream several years earlier. However, the causative factor of that shock was an allergy to lupin and was not caused by ingestion of vanilla, which is suggested by the title of this publication and is misleading to the reader.

## 4. Vanillin in E-Cigarettes as an Unexpected Factor Contributing to the Development of Asthma and Other Organ and Tissue Dysfunctions

In recent years, there has been a preference for replacing traditional cigarettes with devices that deliver nicotine through so-called vaporization. This trend is particularly visible among young people [49]. These devices, including electronic cigarettes (e-cigarettes), are called “Alternative Nicotine Delivery Systems” (ANDS) or “Electronic Nicotine Delivery Systems” (ENDS). The term ENDS was proposed in 2009 by the WHO Study Group on Tobacco Regulation [50,51]. Vaporization is the process of heating dried plant material or concentrate (including liquid) to a temperature that releases active substances (e.g., nicotine) in the form of vapor, without combustion. This smokeless nicotine delivery technique is considered a safer method of delivering nicotine than traditional tobacco smoking [49,51].

The first smokeless non-tobacco cigarette was patented by H.A. Gilbert in 1965 in the USA [52]. Gilbert’s e-cigarette used a battery-powered heater and a liquid cartridge with the active ingredient. This device allowed the user to inhale warm, flavored vapor into the mouth or lungs [52]. Although Gilbert’s device was effective, it failed to gain acceptance among early consumers. It was not until 2013 that Chinese pharmacist Hon Lik patented an electronic atomizing cigarette, which consisted of a battery-powered device for vaporizing a liquid solution of propylene glycol and vegetable glycerin in which nicotine was dissolved [53]. Electronic cigarettes (ECs) are battery-powered devices that heat and atomize a liquid solution, usually containing nicotine. The e-cigarette liquid typically consists of four basic ingredients: propylene glycol (PG), vegetable glycerin (VG), food flavorings, and an active ingredient. The active ingredient is usually nicotine, although other active ingredients can be delivered using this technique, and e-cigarettes are also available without any active ingredient [54]. The latter are referred to as electronic non-nicotine delivery systems (ENNDS) and use cartridges containing all ingredients except the active substance [50].

Vaporization is considered a safer alternative to nicotine delivery than traditional cigarettes [55] and an effective way to quit smoking [51]. However, there have been reports in the literature of lung damage associated with the use of e-cigarettes or vaping products (so-called e-cigarette vaporizer-associated lung injury; EVALI) [56,57]. According to available research, the negative impact of flavorings used in e-cigarettes is not limited to the respiratory tract but may also affect other tissues and organs. According to the analysis by Sachdeva et al. [58], flavorings may have immunomodulatory effects, promoting inflammation, oxidative stress, DNA damage, dysfunction, and electrophysiological changes in the endothelium, leading to epithelial barrier disruption [58].

The pathophysiology of vaping-induced lung injury is still under investigation. Clapp and Jaspers [55] point out that comparisons of the toxicity of traditional cigarettes and e-cigarettes often focus on analyzing the effects of typical cigarette smoke toxicants and known pathologies associated with tobacco smoking. This research strategy does not consider the effects of e-cigarette-specific substances (e.g., flavorings) and the unique effects of the delivery technique (vaporization). Both of these factors likely produce different respiratory effects than those observed in cigarette smokers. It is likely that the key factor in e-cigarette toxicity is exposure of the respiratory tract to vapors of substances other than nicotine, including flavors [55].

Vanillin is one of the most commonly used flavorings in e-cigarette liquids [59]. It is present in 35% of all e-cigarette liquids [59,60]. O-vanillin and other flavoring compounds (e.g., diacetyl, acetylpropionyl, maltol) have been shown to induce local production of reactive oxygen species (ROS) and proinflammatory interleukin-8 (IL-8) in lung tissue, thereby promoting inflammation that can lead to impaired lung function [59].

Muthumalage et al. [61] also observed that exposure of lung epithelial cells (16-HBE and BEAS-2B cell lines) and monocytes (U937 cell line) to various flavored e-liquids stimulated the secretion of inflammatory mediators such as IL-8 or prostaglandin E2 (PGE2), epithelial dysfunction (16-HBE cells), increased oxygen free radical production (16-HBE and BEAS-2B cells), and DNA damage in monocytes. Gerloff et al. [62] observed that flavoring substances (acetoin (butter), diacetyl, pentanedione, maltol (malt), ortho-vanillin (vanilla), coumarin, and cinnamaldehyde) rapidly disrupted epithelial barrier function in human bronchial epithelial cells (16-HBE), while o-vanillin significantly stimulated IL-8 release in bronchial epithelial cells (Beas2).

**Figure 4:**
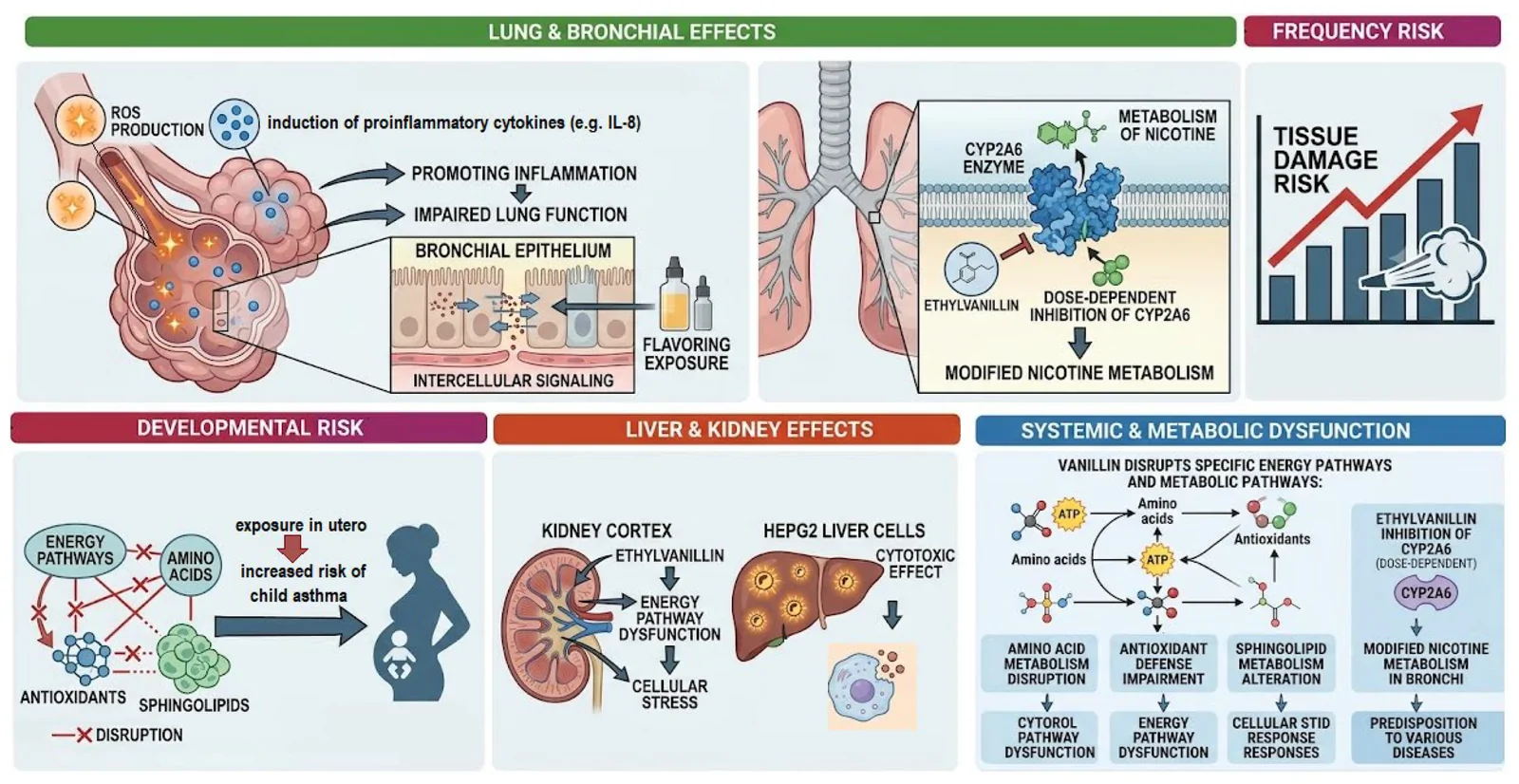
Vanillin in e-cigarette liquids—risks and potential damage [56,57,58,59,60,61,62,63,64,65,66,67,68,69].

Studies by Sherwood et al. [63] revealed that exposure to vanillin and other flavorings from e-cigarette liquids disrupts intercellular signaling in the bronchial epithelium. Furthermore, Winter et al. [64] demonstrated that some e-cigarette flavorings, including ethylvanillin, exhibit dose-dependent inhibition of the cellular enzyme CYP2A6 (the main nicotine-metabolizing enzyme) and thus may potentially modify nicotine metabolism in the bronchial tree. Smith et al. [65] analyzed the effect of vanillin from e-cigarettes on the metabolome of human bronchial epithelium. They demonstrated that vanillin disrupts specific energy pathways and pathways of amino acid, antioxidant, and sphingolipid metabolism, and that changes in these pathways may predispose to the development of various diseases. These researchers believe that vanillin contained in e-cigarettes may be significantly associated with the development of respiratory diseases in individuals exposed to it. Noël et al. [66] also demonstrated in an animal model that exposure to vanilla-flavored e-cigarettes in utero increases susceptibility to the development of asthma later in life. Available literature data suggest that vaping chemicals, including flavorings, in e-cigarette liquids may also have toxic effects on other tissues and organs. For example, Cox et al. [67] demonstrated that the flavoring ethyl vanillin (ETH VAN) can cause energy pathway dysfunction and cellular stress responses in a renal model, while Ricard et al. [68] demonstrated that vanillin, ethylvanillin, and ethylmaltol reduce the viability of HepG2 liver cells. Furthermore, these researchers found that repeated, multiple exposures of HepG2 cells to these flavors enhanced the cytotoxic effect, suggesting that the risk of tissue damage increases with increasing vaping frequency [68] (Figure 4).

The e-cigarette liquids tested in this study are composed of multiple ingredients, including vanillin, and although it is suspected that flavorings may play a significant role, the specific factor responsible for the observed changes has not been identified [61]. According to research by Jabba et al. [69], it is possible that the toxic effects of e-cigarettes are not dependent on a single component of vaping liquids, but on the reaction products between these components. These researchers noted that the reaction products of flavor additives and solvents formed in e-liquids have different toxicological properties than pure flavors.

However, conclusions regarding the toxic effects of vanillin vaping on the respiratory tract are inconsistent. There are also studies that do not confirm a harmful effect of this e-cigarette liquid flavor. According to Emma et al. [70], ethanol used as a solvent, rather than vanillin, is responsible for endothelial cell dysfunction caused by vaping. These researchers, analyzing the effects of ethanol-based vanillin-flavored e-cigarette liquid vapors, did not observe a pro-inflammatory effect of vanillin on ICAM-1 and IL-6 gene expression. Vanillin, unlike ethanol, also had no effect on the viability of the exposed cells (human aortic endothelial cells) or their ability to produce free oxygen radicals [70].

## 5. Summary

Vanillin is primarily responsible for the characteristic flavor and aroma of vanilla. Vanillin, vanillin, and its derivatives are the most popular flavorings used in foods, medicines, cosmetics, e-cigarettes, and other flavored pharmaceutical, food, and industrial products. It is likely that both vanilla and vanillin can induce hypersensitivity reactions and have toxic, irritating, and pro-inflammatory effects. The mechanism of development of hypersensitivity to vanillin/vanillin has not yet been clearly elucidated. The etiopathogenetic patterns of other harmful effects of this flavor have also not been definitively determined.

The clinical course of reported allergic reactions, particularly the limited range of lesions and the long time from exposure to the onset of first symptoms, suggests type IV hypersensitivity reactions in which vanillin is the hapten. Clinically, hypersensitivity to vanilla and/or vanillin most often manifests as a limited rash (of various types) associated with itching and/or swelling. Exacerbation of asthma or atopic dermatitis has also been observed. Symptoms can occur after contact with food or airborne contact. It is worth noting that vanillin may also be an occupational allergen in individuals chronically and systematically exposed to this substance in the work environment through contact and/or inhalation, especially if exposure to vanillin or its synthesis products is accompanied by additional irritants, including mechanical ones (e.g., dust). Hypersensitivity to vanilla and/or vanillin appears rare, but it is also possible that due to limited diagnostic capabilities and the lack of new research, this phenomenon is likely greatly underestimated.

There are also hypotheses that vanillin and other flavorings and fragrances may be new, as-yet poorly studied, factors damaging the respiratory tract after inhaling e-cigarette vapor (vaping), which may contribute to the development or exacerbation of asthma. There is also a hypothesis that e-cigarette use by pregnant women may promote the development of asthma in children later in life. It is also suspected that the toxic effects may also extend to distant tissues and organs (e.g., the liver or kidneys). This may be a significant, albeit little-known, harmful factor associated with this type of exposure, with significant public health implications. However, the currently available literature on the effects of e-cigarette flavors is very limited, and the results of available studies are not always consistent. This significantly complicates reliable conclusions. This highlights the need for further analysis and research on this topic.

## Data Availability

No new data were created or analyzed in this study. Data sharing is not applicable to this article.
